# In Vitro Monitoring of Taste Compound Release During Chewing According to Process‐Related Parameters of the Food

**DOI:** 10.1111/jtxs.70053

**Published:** 2025-11-30

**Authors:** Raphaël Monod, Ana Carolina Conti, Emmanuel Denimal, Chantal Septier, Bérénice Houinsou Houssou, Hélène Brignot, Rohit Srivastava, Sylvie Clerjon, Hélène Labouré, Thierry Thomas‐Danguin, Christian Salles

**Affiliations:** ^1^ Université Bourgogne Europe, Institut Agro, CNRS, INRAE, UMR CSGA Dijon France; ^2^ UR QuaPA Université Clermont Auvergne, INRAE Saint‐Genès‐Champanelle France; ^3^ Department of Food Engineering and Technology São Paulo State University (Unesp), Institute of Biosciences, Humanities and Exact Sciences (Ibilce), campus São José Do Rio Preto São Paulo Brazil; ^4^ Institut Agro Dijon France; ^5^ INRAE PROBE Research Infrastructure, AgroResonance Facility St Genes Champanelle France

**Keywords:** chewing simulator, food breakdown, glutamate, oral processing, release, salt

## Abstract

During consumption, the breakdown of food in the mouth results in the release of molecules responsible for flavor perception. The mechanisms underlying this release, as well as the progressive formation of the food bolus, are strongly influenced by both the composition of the food and the individual's oral physiology. This study focuses on the release of nonvolatile taste compounds under oral conditions. To investigate these mechanisms under controlled conditions, we utilized an in vitro device capable of simulating key oral functions. Two applications illustrating taste compound release during food oral processing demonstrate the device's capabilities. The first application explores the relationships between the processing conditions of extruded corn grits, the properties of the food bolus, and the release of taste compounds (specifically sodium and glutamate ions). Artificial saliva and food bolus samples were collected at various chewing times. The release of taste compounds was found to be influenced by the composition of the food matrix; notably, the presence of oil led to greater releases of sodium and glutamate. The second application involves cooked carrots and salt application practices, with the aim of optimizing discretionary salt intake while maintaining an acceptable perception of saltiness. Different types of salt (Fleur de sel and fine sea salt) were added either during or after cooking. An inhomogeneous distribution of salt on the surface of cooked carrots resulted in a brief but intense sodium release, which may explain the increased perception of saltiness when salt is applied after cooking.

## Graphical abstract

Two applications of in vitro studies on taste compound release and food bolus formation using a chewing simulator are presented. The addition of oil appears to promote the release of taste compounds from corn grit snacks. For cooked carrots, the greater variability and inhomogeneous distribution observed with the plate salting method account for the more intense sodium release, which may be associated with a stronger in vivo perception of saltiness.

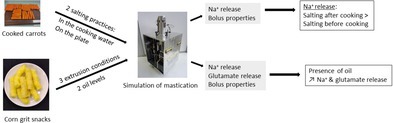

## 1 Introduction

During consumption, the breakdown of food in the oral cavity leads to the release of molecules responsible for flavor perception. However, this phenomenon is highly complex. Oral processing of food involves several operations, including mastication, intra‐oral transportation, saliva impregnation, progressive reduction in particle size, formation of a cohesive bolus, and swallowing (J. S. Chen 2009). Simultaneously, volatile compounds responsible for aroma perception and nonvolatile compounds responsible for taste perception are progressively released: volatile compounds enter the gas phase, while nonvolatile compounds are dissolved in saliva within the oral cavity (J. Chen 2015; De Lavergne et al. 2021; Salles et al. 2011).

The mechanisms involved in the release process and the progressive formation of the food bolus are strongly linked to food composition, texture, individual chewing parameters, and salivation (Guichard et al. 2023). Oral and structural parameters are often intricately intertwined. For example, different salt release and saltiness perception profiles have been observed and are explained by physiological differences among subjects (Phan et al. 2008; Pionnier, Chabanet, et al. 2004; Pionnier, Nicklaus, et al. 2004). These studies found that higher water content in model cheeses increased sodium release at the beginning of chewing, when the saliva‐to‐food ratio was still low and only the water present in the product contributed to sodium release. Furthermore, it was only at high water content that a high fat content further enhanced sodium release, suggesting noteworthy interactions between fat and water in the in‐mouth release of sodium. Regardless of the mechanism, differences in chewing behavior between subjects were reported to explain most of the variability in sodium release and saltiness perception in the oral cavity. Subjects exhibiting higher chewing force and lower salivary flow rate experienced greater sodium release and heightened saltiness intensity (Lawrence, Buchin, et al. 2012; Lawrence, Septier, et al. 2012).

To address the limitations of flavor compound release studies in human subjects—such as inter‐individual variability, moderate intra‐individual reproducibility, and the necessity for good acceptability of food samples by panelists—chewing devices have been developed to simulate certain functionalities of oral food processing in vitro (Panda et al. 2020; Salles and Benjamin 2017). In particular, such devices have been successfully employed to study the release of volatile compounds from solid food matrices under controlled and decoupled oral parameters, demonstrating the influence of each parameter on the temporal release profile of individual volatiles (Benjamin et al. 2012; Hayashi et al. 2022; Tarrega et al. 2019). Food bolus formation has also been investigated under in vitro controlled oral conditions. For example, the fragmentation, moisture content, and viscosity of chewed extruded pea snacks were correlated with the structural characteristics of the raw product. This relationship enables modeling of oral processing phenomena for brittle extruded products and facilitates the design of extruded vegetables with targeted structures via reverse engineering based on oral processing mechanism models (Kristiawan et al. 2020). Additionally, another study demonstrated that plant‐ and beef‐based patties can be differentiated during mastication by differences in granulometry and texture exhibited in the formed boluses (Giron et al. 2025).

To our knowledge, no dynamic in vitro study investigating the release of taste compounds under oral conditions has been reported to date for food products. Such studies are highly relevant because, on one hand, taste perception is a crucial factor influencing consumer acceptability (Guichard and Salles 2023), and on the other hand, certain taste stimuli, such as salt (He et al. 2020) and sugar (Cara et al. 2024), pose significant health risks when consumed in excess. This underscores the need to reformulate products with reduced salt and sugar levels and to comprehensively assess the impact of these reformulations on all food properties to ensure that consumer acceptability is maintained.

To investigate these mechanisms under controlled conditions, we developed an in vitro device capable of simulating oral functions and enabling saliva sampling throughout the oral processing. The objective of our study was to demonstrate two applications illustrating the device's capability for in vitro investigations of taste compound release and food bolus formation.

## 2 Materials and Methods

### 2.1 Chewing Simulator Device

**FIGURE 1:**
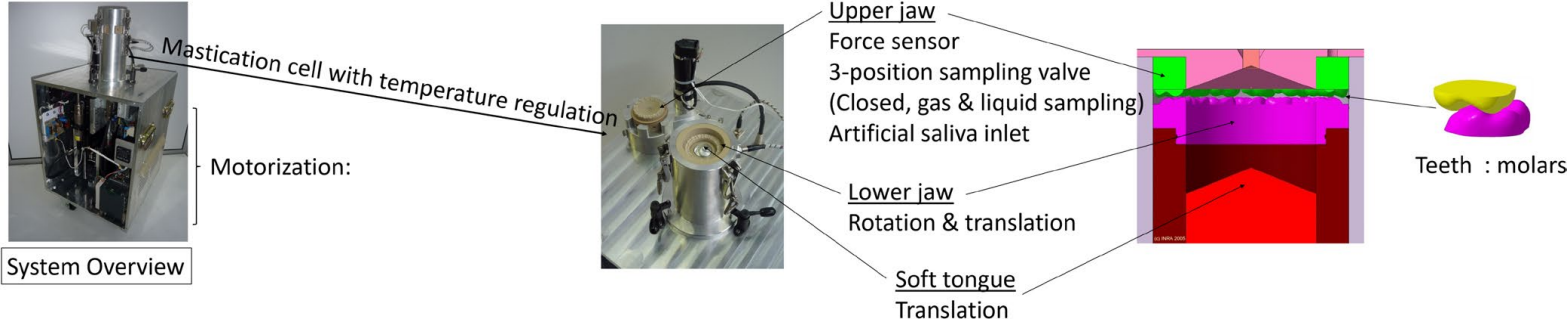
Chewing simulator used in the study.

A new chewing simulator (BA2), developed at CSGA in Dijon and based on a previously developed device (Mielle et al. 2010; Salles et al. 2007) with the same functionalities but several improvements, was used to monitor sodium and glutamate release as well as bolus formation during in vitro mastication of foods (Figure 1).

It replicates compression and shearing forces generated by jaw and tongue movements, surface morphology, and salivary flow rate. Specifically, the forces exerted by the jaws and tongue are regulated using a force sensor, and the tongue is equipped with an interchangeable soft membrane at its tip. A user‐friendly software interface controls all the setting parameters.

BA2 applies controlled compression and shear forces to the food sample using fixed upper and mobile lower jaws equipped with molar‐type teeth, and features a flexible, mobile tongue that compresses the palate, all under a continuous flow of artificial saliva. Artificial saliva used in the present study was made of NaHCO_3_: 5.208 g/L, K_2_HPO_4_ (3H_2_O): 1.369 g/L, NaCl: 0.877 g/L, KCl: 0.477 g/L, CaCl_2_ (2H_2_O): 0.441 g/L, Mucine from porcine pancreas: 2.16 g/L, Alpha‐amylases from porcine pancreas: 6.7 g/L (Odake et al. 2000).

The system is driven by linear motors. This device closely mimics human mastication under realistic conditions, accounting for volume, temperature, cycle duration, applied forces, and number of chews, as already reported in our previous works (Kristiawan et al. 2020; Mielle et al. 2010; Salles et al. 2007; Tarrega et al. 2019). The experimental procedure employed in the chewing simulator was specifically adjusted according to oral parameters determined through preliminary in vivo experiments conducted with subjects consuming the same food products. This calibration was subsequently fine‐tuned based on the visual and textural characteristics of the resulting bolus, depending on its consistency and the specific objectives of the study. A force sensor located above the upper jaw and palate enables precise measurement of the forces applied alternately by the lower jaw and the tongue, facilitating improved motor control. Both gaseous and liquid phases can be sampled during the experiment. In particular, the liquid phase can be aliquoted during the process for offline physicochemical analyses, allowing investigation of the release of target compounds during a controlled oral processing simulation.

### 2.2 Samples Preparation

#### 2.2.1 Corn Grits

Corn grits (Master SP Alimentos, Capela do Alto, Brazil) were initially adjusted to moisture contents of 10%, 15%, and 20% (dry basis) using pure water. Subsequently, cysteine (0.2 g/100 g, L‐cysteine HCl anhydrous, purity > 98.6%, Lepuge Insumos Farmacêuticos Ltda., São Bernardo do Campo, Brazil) and butyric acid (0.4 g/100 g, purity > 99%, Sigma‐Aldrich, Milwaukee, USA) were added as aroma precursors of cheese (Menis‐Henrique et al. 2020). These ingredients were mixed by manual agitation in polyethylene bags and refrigerated for 24 h after sealing. Prior to extrusion, the mixture was equilibrated at room temperature for 2 h.

**FIGURE 2:**
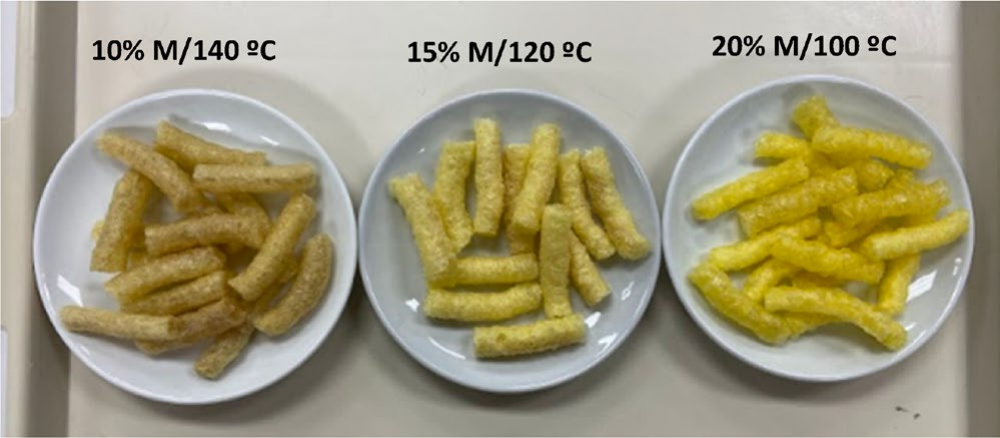
Pictures of corn snacks obtained under different extrusion conditions. M, moisture content of corn grits (dry basis) before extrusion. Temperature in zone 5 of the extruder.

The corn grits were extruded in an RXPQ Labor 24 single screw extruder (INBRAMAQ, Ribeirão Preto, Brazil) with five independent heating zones, under the following conditions: helicoidally grooved barrel; screw with a large step, one exit, with a compression ratio of 3.3:1 and length‐to‐diameter ratio of 15.5:1; pre‐die extruder with holes of 2.7 mm; extruder die with a diameter of 2.9 mm (round hole); feed rate of 265 g/min; screw speed at 192 rpm; temperatures in zones 1 to 3: off (approximately 40°C), 70°C and 90°C, respectively. Finally, the three extrusion conditions were: 20% moisture (M)/100°C; 15% moisture/120°C; and 10% moisture/140°C. Pictures of corn grits that have undergone these three extrusion conditions are shown in Figure 2.

After extrusion, samples of approximately 5 cm in length were cut using a mold and supplemented with salt (1.0 g/100 g, Cisne, Cabo Frio, Brazil) and monosodium glutamate (0.4 g/100 g, Ajinomoto, Limeira, Brazil) (Menis‐Henrique et al. 2020). Each combination of moisture and temperature was divided into two portions: one without added sunflower oil, and the other sprinkled with sunflower oil (Liza, Mairinque, Brazil) so that the final lipid content was 7.5 ± 0.15 g of lipids per 100 g (analyzed in the laboratory). The parameters of the chewing simulator were adjusted as follows: bite force: 300 N; initial artificial saliva volume: 4.5 mL; artificial salivary flow rate: 5 mL/min; chew cycle numbers: 4, 8, and 12; temperature: 35°C.

#### 2.2.2 Cooked Carrots

**FIGURE 3:**
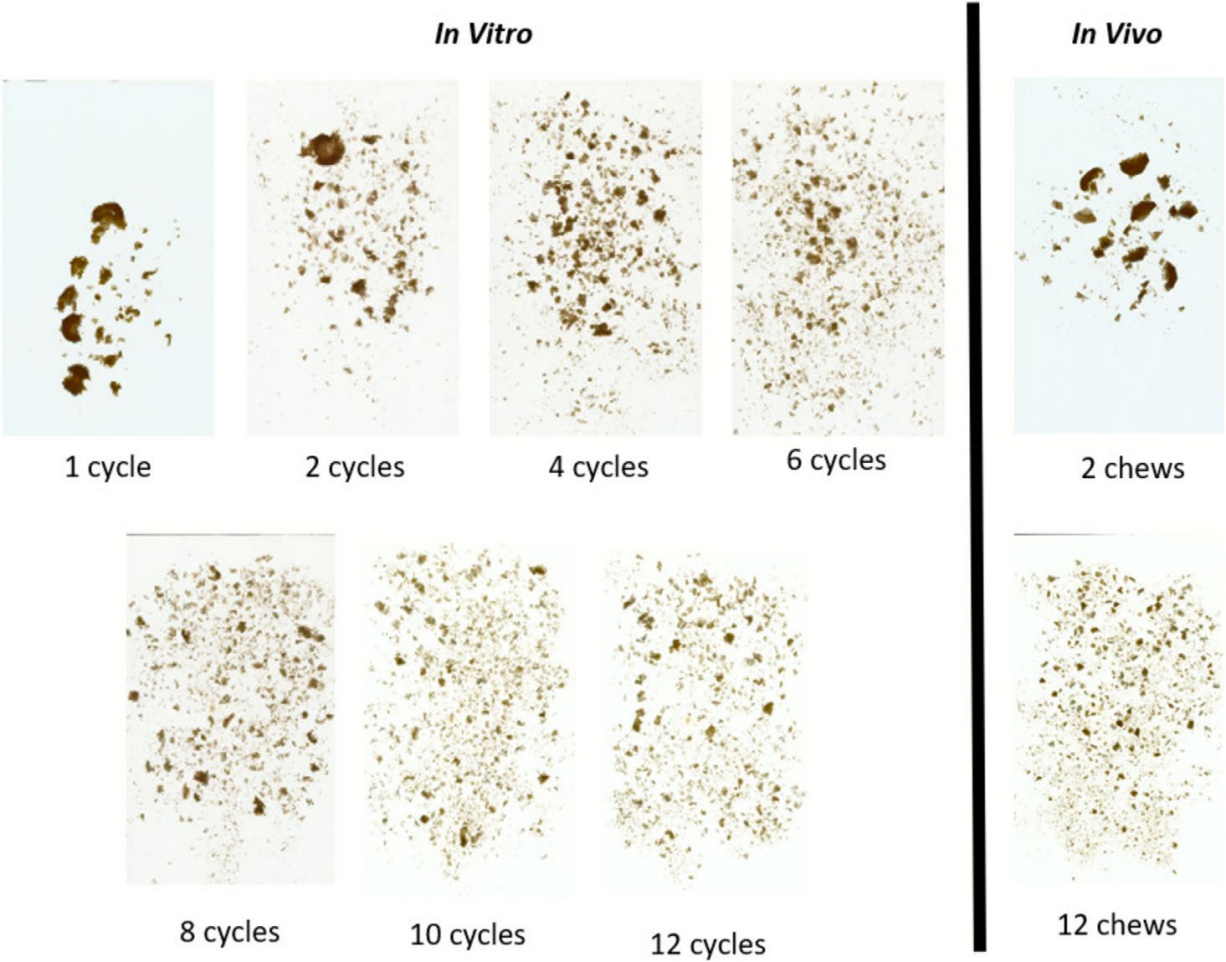
In vitro and in vivo bolus after a certain number of mastication cycles or chews of cooked carrots.

Cooked carrots were prepared following a previously described specific procedure (Monod et al. 2024). The parameters of the chewing simulator were adjusted to produce an in vitro bolus representative of the in vivo bolus. This adjustment was based on the overall visual characteristics of the bolus observed during preliminary tests. The boluses obtained from in vitro and in vivo mastication are illustrated in Figure 3.

Salt was added either in water at the beginning of the cooking process or directly onto the cooked and sliced carrots after cooking. A single salt concentration was used: 10 g/L in the cooking water (referred to as the “regular level”). This regular level was selected to match the typical amount found in recipes for cooking carrots in water and was confirmed through an internal preliminary test to elicit a Just‐About‐Right perception of saltiness.

After cooking and slicing, the carrot slices were stored in stainless steel trays for approximately 30 min before the first sample was placed in the chewing simulator, and up to 75 min before the last sample. Carrots requiring salt addition on the plate were weighed (*m* = 87.6 ± 5.2 g) to determine the amount of salt to be added, aiming to obtain the same salt content as carrots cooked in salted water. This quantity was estimated based on sodium content measured by ion chromatography in preliminary analyses (Monod et al. 2024). After salt addition, the carrot slices were gently mixed with a spoon for 15 s. A sample consisted of three carrot slices (*m* = 4.3 ± 0.3 g), which were then placed in the chewing simulator.

Preliminary in vivo mastication tests indicated that the number of chews before swallowing was approximately 12. In vitro mastication under a chewing force of 150 N showed that after 10 and 12 cycles, the food bolus was visually comparable to the in vivo bolus just prior to swallowing. Additionally, the bolus obtained after one in vitro chew resembled the bolus after two chews in vivo. Therefore, we chose to investigate a range of in vitro chewing cycles from 1 to 10.

The experimental conditions were set as follows: chewing and tongue force of 150 N; no shear force applied; food sample mass of 4.3 ± 0.3 g; artificial saliva volume of 2 ± 0.01 mL; chewing cycle duration of 2 s; and temperature maintained at 33°C. The numbers of masticatory cycles tested were 1, 2, 3, 4, 5, 6, 8, and 10. For each chewing cycle, the entire carrot bolus was removed from the chewing simulator cell to recover the liquid phase. Thus, a new carrot sample is used for each chewing time.

### 2.3 Water Phase Sampling and Analyses

To determine sodium and glutamate concentrations, the liquid phase was directly sampled from the bolus and centrifuged to remove particles. For each product and each mastication cycle number, three replicates were performed. Between 0.2 and 1.0 mL of sample was collected for quantification. Sodium ion concentration was measured by ion chromatography with conductivity detection (Conti, Septier, Gourrat, et al. 2025).

Glutamate concentration was quantified using a commercial biochemical kit (STA‐674, Cell Biolabs, San Diego, CA), with assays performed in duplicate. Quantification was based on enzymatic reactions coupled with fluorescence detection (excitation: 570 nm; emission: 585 nm). Data were recorded using a microplate reader (Ensight, PerkinElmer, Waltham, MA) and analyzed following an internal quality control procedure.

### 2.4 Statistics

All data related to the corn grits were analyzed using XLSTAT statistical software for Microsoft Excel (Addinsoft, New York, USA) with a significance level set at 0.05. The release of compounds as a function of the number of chews was modeled using simple linear regression. Additionally, the data were subjected to analysis of variance (ANOVA) with extrusion condition, oil addition, and number of chews as factors, including their two‐way interactions, followed by a Wilcoxon test.

For the carrot study, all data analyses were performed using RStudio software version 4.1.2 (R Core Team 2021). Linear models were fitted using the lm function from the stats R package. One‐way ANOVAs were conducted using the ANOVA function from the rstatix package (v0.7.0; Kassambra 2021). Tukey post hoc tests with false discovery rate (FDR) correction were applied when the ANOVA *p*‐value was below 0.05.

## 3 Results and Discussion

### 3.1 Corn Grits

**FIGURE 4:**
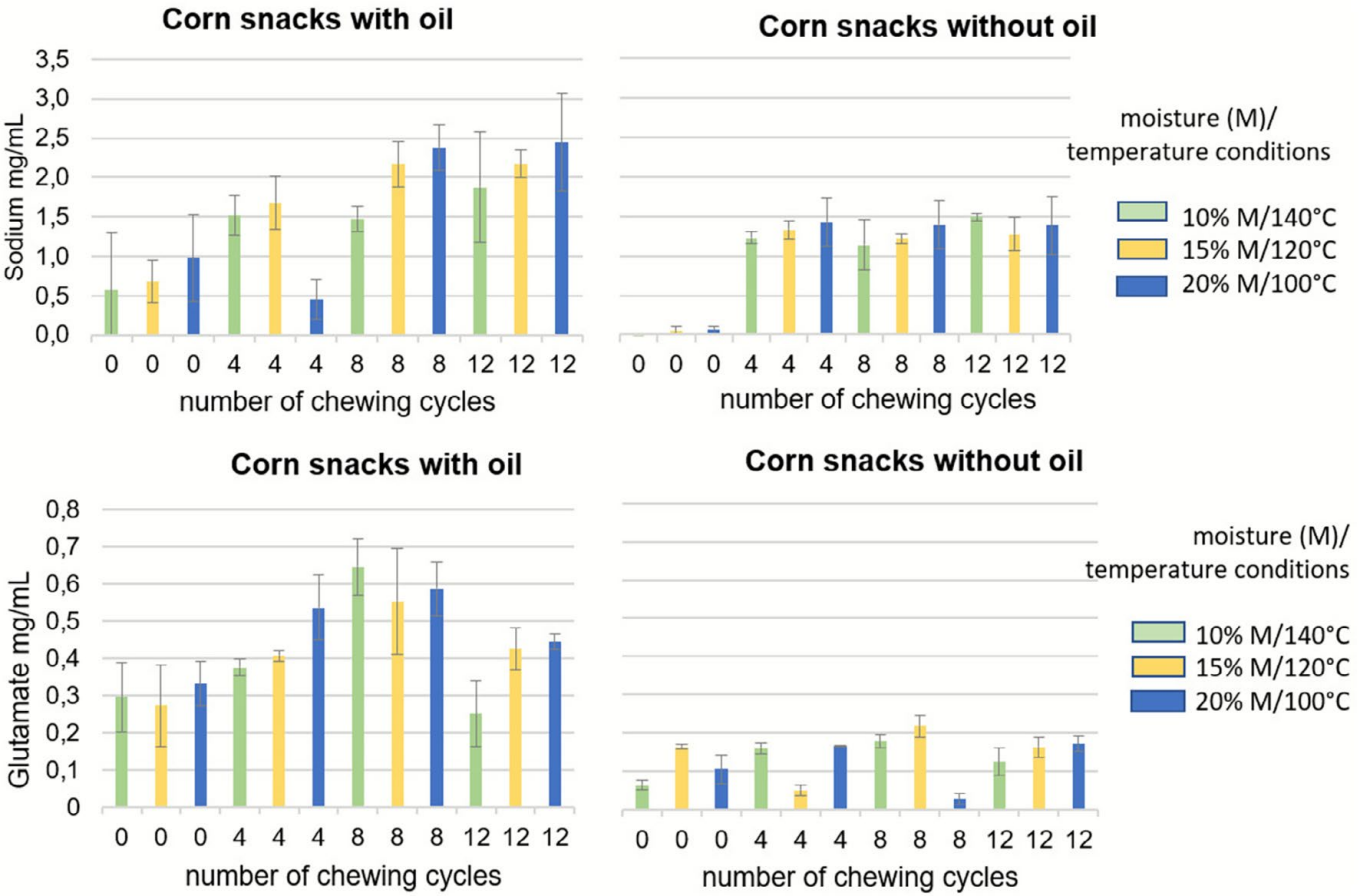
Sodium and glutamate release results for the corn grit snacks with and without oil in the three extrusion conditions. Bars indicate the standard deviations (*n* = 3).

The moisture contents of the corn grits (wet basis): 7.0 g/100 g, 7.7 g/100 g, and 7.7 g/100 g for 10% M/140°C, 15% M/120°C, and 20% M/100°C, respectively, were rather close despite the different extrusion conditions. Corn grit snacks produced under three extrusion conditions and two fat levels (0 and 7.5 ± 0.15 g/100 g) were subjected to chewing simulation to investigate the relationships between processing conditions of extruded corn grits, bolus properties, and the release of taste compounds (sodium and glutamate ions) during oral processing simulated with the chewing simulator. The main taste compounds in these products are sodium chloride and glutamate, which are responsible for salty and umami tastes, respectively. The release results are presented in Figure 4.

As expected, considering all extrusion conditions combined, the linear regression model explained the release of sodium as a function of the number of chewing cycles, both in the presence (*R* ^2^ = 0.443, *p* < 0.0001) and absence (*R* ^2^ = 0.541, *p* < 0.0001) of oil. Similar results were observed for each condition without oil: 10% moisture/140°C (*R* ^2^ = 0.550, *p* = 0.014), 15% moisture/120°C (*R* ^2^ = 0.546, *p* = 0.006), and 20% moisture/100°C (*R* ^2^ = 0.530, *p* = 0.007). When oil was added, the model was significantly explanatory for 10% moisture/140°C (R^2^ = 0.629, *p* = 0.002) and 15% moisture/120°C (*R* ^2^ = 0.779, *p* < 0.0001), but not for the 20% moisture/100°C condition (*R* ^2^ = 0.306, *p* = 0.062). Since oil was sprayed onto the surface after extrusion, the lack of significance in this latter case may be attributed to the specific distribution of oil droplets resulting from the product structure induced by extrusion conditions. This hypothesis warrants further verification through additional structural analyses, as differences in salt release were already reported according to differences in food structure (Benjamin et al. 2018).

**FIGURE 5:**
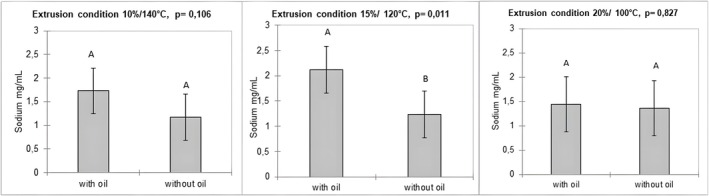
Sodium release from corn grits extruded under three moisture (M)/temperature conditions, with and without oil (each condition treated separately) for all chewing cycles combined. Different letters indicate different statistical means according to the Tukey test (*p* < 0.05). Bars indicate the standard deviations (*n* = 3).

The release of sodium was also influenced by the presence or absence of oil, with a significantly greater release observed in the presence of oil only under the 15% moisture/120°C extrusion condition (*p* < 0.011) (Figure 5).

Regarding glutamate, the linear regression model explained glutamate release as a function of the number of chewing cycles for the three extrusion conditions in corn grits without oil: 10% moisture/140°C (*R* ^2^ = 0.295, *p* = 0.002), 15% moisture/120°C (*R* ^2^ = 0.781, *p* < 0.0001), and 20% moisture/100°C (*R* ^2^ = 0.669, *p* = 0.001). For corn grits with oil, the data exhibited greater variability, and the model did not significantly explain glutamate release under any extrusion condition: 10% moisture/140°C (*R* ^2^ = 0.124, *p* = 0.261), 15% moisture/120°C (*R* ^2^ = 0.211, *p* = 0.133), and 20% moisture/100°C (*R* ^2^ = 0.042, *p* = 0.524).

**FIGURE 6:**
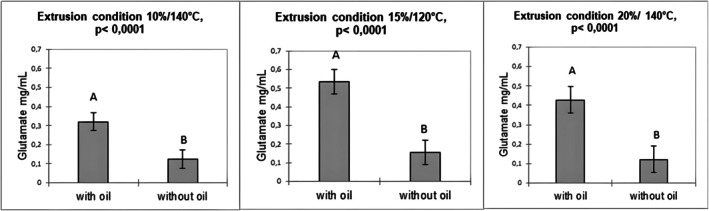
Glutamate release from corn grits extruded under three moisture (M)/temperature conditions, with and without oil (each condition treated separately) for all chewing cycles combined. Different letters indicate different statistical means according to the Tukey test (*p* < 0.05). Bars indicate the standard deviations (*n* = 3).

The release of glutamate is also influenced by the presence or absence of oil, with more pronounced effects. Glutamate release was significantly greater in the presence of oil across all three extrusion conditions (*p* < 0.0001) (Figure 6).

**FIGURE 7:**
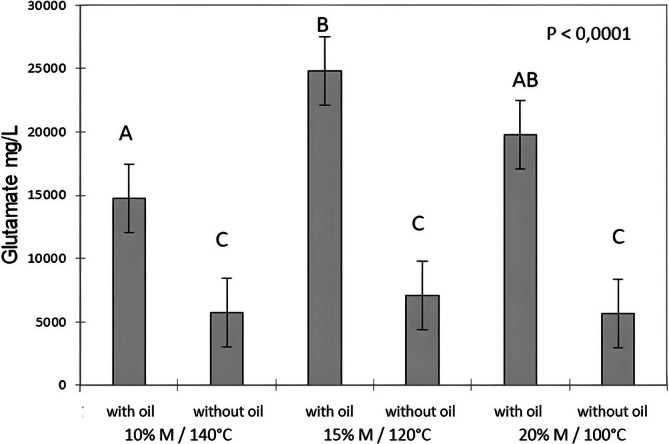
Glutamate release from corn grits extruded under three moisture (M)/temperature conditions, with and without oil. Different letters indicate different statistical means according to the Tukey test (*p* < 0.05). Bars indicate the standard deviations (*n* = 3).

When considering only the oil content factor across the three extrusion conditions, a highly significant effect of oil content on glutamate release was observed (*p* < 0.0001) (Figure 7). The presence of oil enhances glutamate release under mastication conditions. Among the three conditions, the 15% moisture/120°C extrusion condition appears most favorable for maximizing glutamate release and is also the mildest processing condition.

Thus, the addition of oil appears to promote the release of taste compounds. The effects of other factors, such as moisture content during extrusion and the number of chewing cycles, on taste compound release are less clear and require further analysis. To our knowledge, no studies have specifically investigated the release of taste compounds such as glutamate or sodium from corn products in the oral cavity. However, an in vivo study conducted on flavored textured soy proteins reported findings that seem to suggest the opposite. Specifically, lower sodium and glutamic acid concentrations were detected in the saliva of panelists when vegetable oil was added, depending on extrusion conditions (Conti, Septier, Gourrat, et al. 2025). The authors reported changes in the texture and physical properties of particles according to extrusion conditions. In particular, they observed that the addition of oil resulted in boluses with lower moisture content and fewer, less uniform particles (Conti, Septier, Denimal, et al. 2025), which could explain these differences in the release of flavor compounds in the mouth. Regarding corn grits, the relationships between particle‐related parameters and factors such as oil presence or absence, extrusion conditions, and the kinetics of compound release are not straightforward. To explain this phenomenon, one explanation could be put forward. The bolus obtained with the corn grits was very compact, very sticky and therefore the particles were probably very prone to re‐agglomeration between them. We can hypothesize that the addition of fat may have a decompaction effect and make the particles of the bolus softer, which would be in line with the promotion of the release of sodium and glutamate in the saliva. This hypothesis remains to be verified. This study requires more in‐depth analyses and integration of additional parameters related to product structure, physicochemical characteristics, and sensory properties to better understand the mechanisms underlying the release of taste compounds. These studies are ongoing and will be published soon.

However, it is important to note that contradictory effects of fat on sodium release and perception have been reported in various studies and summarized in a review (Kuo and Lee 2014). While multiple factors may account for these discrepancies in in vivo studies, within the context of the present in vitro study using a chewing simulator, the influence of matrix composition is the most plausible explanation, although this remains to be demonstrated.

### 3.2 Cooked Carrots

For boiled carrots, salting after cooking with relatively large salt crystals, such as “fleur de sel,” has been shown to enhance salt perception compared to salting in water, during cooking (Monod 2024). Furthermore, when using this type of salt crystal, the sodium distribution was significantly more heterogeneous than with salting in water (Monod, Bonny, et al. 2025). Therefore, we hypothesize that this heterogeneous distribution induces a rapid sodium release at the onset of oral processing, which is known to enhance saltiness perception (Busch et al. 2009). In contrast, salting in water results in a more homogeneous sodium distribution, likely producing a slower and more uniform sodium release over time. This mechanism may explain the sensory outcomes reported in a previous study (Monod 2024). The objective of the present in vitro study was to monitor the dynamics of sodium release during simulated mastication of carrots, as a function of salting method, under controlled physiological conditions for the different salting modalities.

**FIGURE 8:**
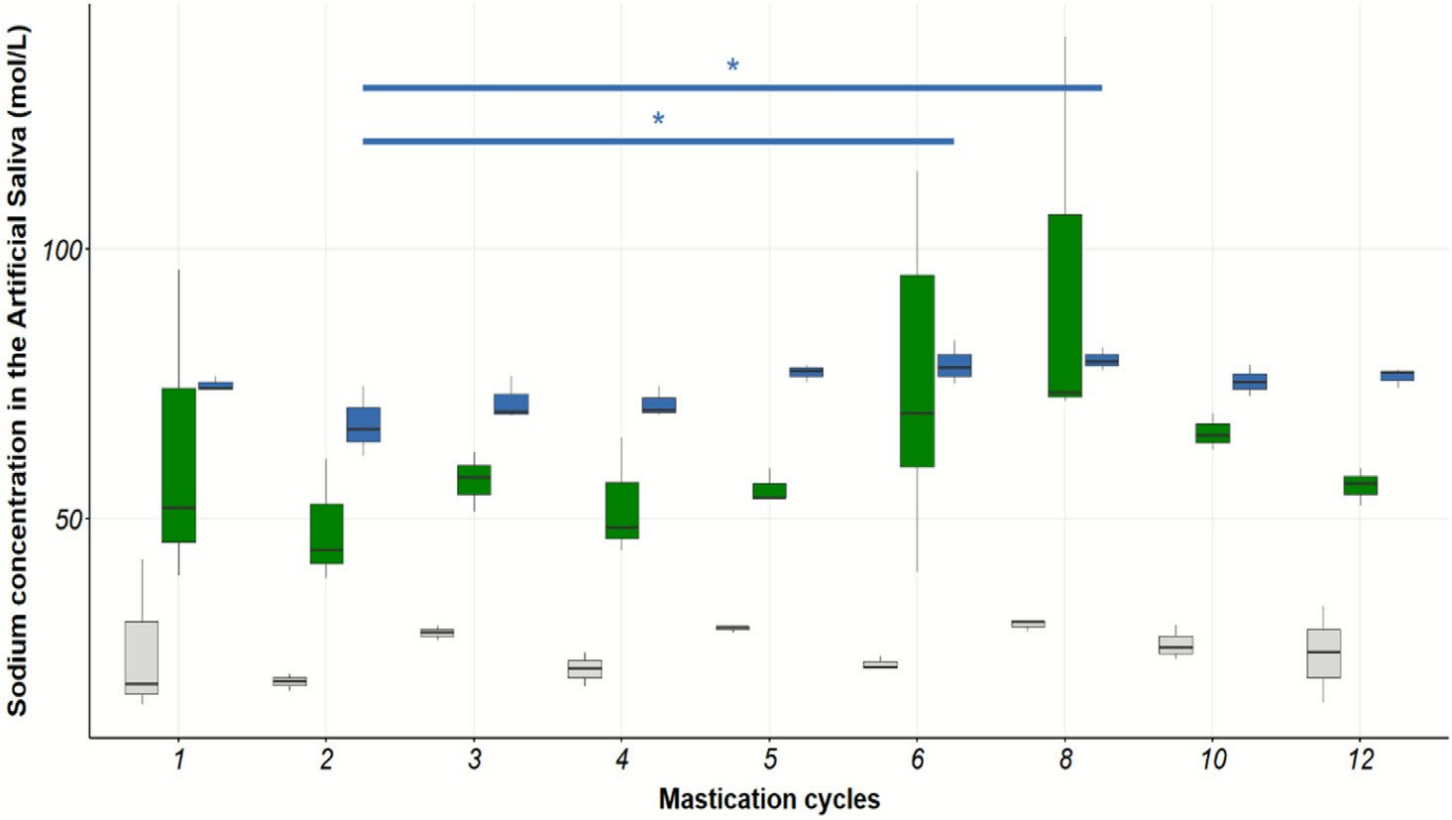
Sodium release from carrots into artificial saliva as a function of the number of cycles applied during the in vitro masticatory process (Boxplots, *n* = 3). Treatments US, FiWReg, and FlPReg are represented by the gray, blue, and green boxes respectively. Significance level: * = *p*‐value < 0.05. US: Unsalted; Fi: Fine sea salt; Fl: « fleur de sel »; W: In water; P: In the plate; Reg: Regular level. Bars indicate the distance from the upper quartile and the lower quartile to the most extreme data points for each box.

Figure 8 shows the sodium content measured by ion chromatography in the artificial saliva samples collected from the BA2 device during mastication of unsalted carrots, carrots salted in water, and carrots salted on the plate with “fleur de sel.” The number of mastication cycles significantly influenced the dynamics of sodium release during mastication when salt was added to water (*F*_(345,7)_ = 3.92, *p* = 0.01). This effect was marginally significant when salt was applied on the plate (*F*_(6383,7)_ = 2.14, *p* = 0.07) and was not significant for unsalted carrots (*F*_(310,7)_ = 1.47, *p* = 0.25). For the salting‐in‐water condition, sodium concentration in the collected artificial saliva was significantly higher at 6 and 8 mastication cycles compared to 2 cycles (*p* < 0.05).

**FIGURE 9:**
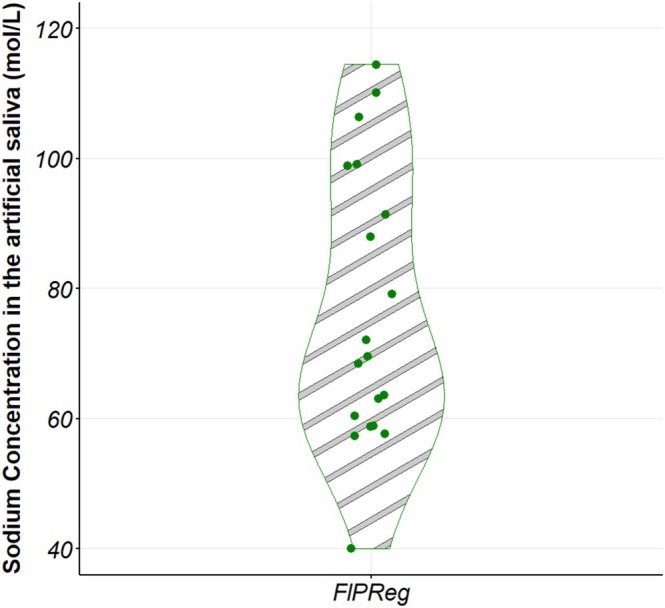
Sodium release after 6 chewing cycles when salting on the plate with fleur de sel (*n* = 19 replicates). Fl: “fleur de sel”; P: In the plate; Reg: Regular level.

A notable feature in Figure 8 is the difference in variability of sodium content between the salting‐in‐water and salting‐on‐the‐plate methods. Accordingly, Figure 9 illustrates the variability of sodium concentration in the artificial saliva after 6 mastication cycles when salting was performed on the plate with fleur de sel. The coefficient of variation for this condition is 27.9% (*n* = 19 replicates). For salting in water, the highest coefficient of variation was observed after two mastication cycles, at 9.6%, which is nearly three times lower than that for salting on the plate with fleur de sel. High variability was also noted after one and eight mastication cycles for the salting‐on‐the‐plate modality, with coefficients of variation of 47.7% and 40.6%, respectively (*n* = 3 replicates).

The amount of sodium released from carrots salted in water increased with the number of mastication cycles. This trend was not significant for the salting‐on‐the‐plate modality or for unsalted carrots. The primary difference between the two salting methods was the variability of sodium release, which was substantially greater when salt was applied on the plate (Figure 8). This increased variability was further confirmed by increasing the number of replicates from 3 to 19 at an intermediate number of chewing cycles (Figure 8). We hypothesize that this variability may cause intermittent bursts of sodium release occurring at specific points during oral processing, likely as early as the first chew. This variability could result from the application method in the salting‐on‐the‐plate modality, where some carrot slices may lack salt crystals while others may have one or more crystals on their surfaces. During mastication in the simulator, it is expected that at certain points, these salt crystals come into contact with saliva and rapidly dissolve, producing transient pulses of sodium ions. This pulse of sodium could explain the heightened saltiness perception, as previously demonstrated (Busch et al. 2009). Such stimulations can result from an inhomogeneous distribution of salt within a food matrix (Noort et al. 2010). An inhomogeneous salt distribution was observed on boiled carrots when salt was poured directly onto the food after cooking, as reported earlier (Monod, Clerjon, et al. 2025). Furthermore, using the same methodology, a 30% reduction in sodium content was achieved without diminishing saltiness perception (Monod 2024). Overall, our results suggest that the heterogeneous distribution of sodium, resulting from salting carrots post‐cooking, leads to a brief but intense sodium release at a specific point during mastication. This is reflected in the high variability of sodium content observed at a given number of mastication cycles and may explain the enhanced saltiness perception associated with the on‐plate salting modality.

## 4 Conclusion

The chewing simulator represents a suitable system for studying temporal flavor release and bolus formation, enabling in vitro investigations that complement and enhance the robustness of in vivo studies. Using two applications involving foods of contrasting textures, this study demonstrates the added value of in vitro approaches for investigating flavor release and food breakdown under simulated mastication conditions. In particular, regarding the release of taste compounds, the use of a chewing simulator has proven to be a relevant tool for exploring various mechanistic aspects.

The lipid content in corn snacks appears to strongly enhance the release of taste compounds during artificial mastication. However, the effects of extrusion conditions and bolus particle morphology on this release phenomenon remain unclear and warrant further investigation. Additionally, salting cooked carrots significantly influences the release of salt into the aqueous phase during mastication. Greater heterogeneity in salt distribution at the food surface may contribute to increased release and, consequently, higher perceived saltiness.

This in vitro approach complements in vivo methods, which, despite being subject to inter‐individual variability, uniquely capture perceptual aspects. Comparing these two approaches is essential for a more comprehensive understanding of oral food processing. This represents a key perspective for future research in the field.

## Author Contributions

**Raphaël Monod:** writing – original draft, writing – review and editing, formal analysis, methodology. **Ana Carolina Conti:** conceptualization, writing – review and editing, methodology, investigation, formal analysis, funding, acquisition. **Emmanuel Denimal:** methodology, formal analysis, data curation. **Chantal Septier:** methodology, data curation, formal analysis. **Bérénice Houinsou Houssou:** methodology, formal analysis, data curation. **Hélène Brignot:** writing – review and editing, methodology, formal analysis, data curation. **Rohit Srivastava:** methodology. **Sylvie Clerjon:** writing – review and editing, methodology, investigation, conceptualization, funding acquisition, supervision. **Hélène Labouré:** funding acquisition, writing – review and editing, investigation, conceptualization, formal analysis, supervision. **Thierry Thomas‐Danguin:** funding acquisition, conceptualization, investigation, project administration, supervision, methodology. **Christian Salles:** writing – original draft, validation, writing – review and editing, project administration, supervision, resources.

## Funding

Cooked carrots study was supported by the French National Research Agency (ANR) [Grant NO. ANR‐19‐CE21‐0009] and the French National Research Institute for Agriculture, Food and Environment (INRAE), Food Bioproducts and Waste Division (TRANSFORM). Corn grit snacks study was supported by Universidade Estadual Paulista ‘Julio De Mesquita Filho’ (UNESP, Sao Paulo, Brazil) and Institut Agro Dijon (France). The development of the chewing simulator was supported by the Regional Council of Burgundy Franche Comté (France) and the SATT SAYENS (Dijon, France).

## Ethics Statement

The authors have nothing to report.

## Conflicts of Interest

The authors declare no conflicts of interest.

## Data Availability

The data that support the findings of this study are available on request from the corresponding author. The data are not publicly available due to privacy or ethical restrictions.
